# Vulnerability of eco-environmental health to climate change: the views of government stakeholders and other specialists in Queensland, Australia

**DOI:** 10.1186/1471-2458-10-441

**Published:** 2010-07-28

**Authors:** Linn B Strand, Shilu Tong, Rosemary Aird, David McRae

**Affiliations:** 1 School of Public Health and Institute of Health and Biomedical Innovation, Queensland University of Technology. Postal address: Victoria Park Rd, Kelvin Grove, Brisbane, QLD 4059, Australia; 2 School of Public Health, Queensland University of Technology. Postal address: Victoria Park Rd, Kelvin Grove, Brisbane, QLD 4059, Australia; 3 Queensland Climate Change Centre of Excellence, Department Of Environment and Resource Management, Queensland Government. Postal Address: 41 George St, Brisbane, QLD 4001, Australia

## Abstract

**Background:**

There is overwhelming scientific evidence that human activities have changed and will continue to change the climate of the Earth. Eco-environmental health, which refers to the interdependencies between ecological systems and population health and well-being, is likely to be significantly influenced by climate change. The aim of this study was to examine perceptions from government stakeholders and other relevant specialists about the threat of climate change, their capacity to deal with it, and how to develop and implement a framework for assessing vulnerability of eco-environmental health to climate change.

**Methods:**

Two focus groups were conducted in Brisbane, Australia with representatives from relevant government agencies, non-governmental organisations, and the industry sector (n = 15) involved in the discussions. The participants were specialists on climate change and public health from governmental agencies, industry, and non-governmental organisations in South-East Queensland.

**Results:**

The specialists perceived climate change to be a threat to eco-environmental health and had substantial knowledge about possible implications and impacts. A range of different methods for assessing vulnerability were suggested by the participants and the complexity of assessment when dealing with multiple hazards was acknowledged. Identified factors influencing vulnerability were perceived to be of a social, physical and/or economic nature. They included population growth, the ageing population with associated declines in general health and changes in the vulnerability of particular geographical areas due to for example, increased coastal development, and financial stress. Education, inter-sectoral collaboration, emergency management (e.g. development of early warning systems), and social networks were all emphasised as a basis for adapting to climate change. To develop a framework, different approaches were discussed for assessing eco-environmental health vulnerability, including literature reviews to examine the components of vulnerability such as natural hazard risk and exposure and to investigate already existing frameworks for assessing vulnerability.

**Conclusion:**

The study has addressed some important questions in regard to government stakeholders and other specialists' views on the threat of climate change and its potential impacts on eco-environmental health. These findings may have implications in climate change and public health decision-making.

## Background

There is wide consensus that human activities have changed and will continue to change the climate of the Earth [1,2]. The Intergovernmental Panel on Climate Change (IPCC) recently stated that the global mean temperature has increased by approximately 0.7 degrees Celsius during the last century, with most of this increase having occurred since the 1970 s [3]. Climate change is argued to be largely caused by an increasing concentration of greenhouse gases in the atmosphere and the global mean temperature is projected to rise a further 1.1 to 6.4 degrees Celsius by 2100, depending on future energy expenditure [3].

Eco-environmental health refers to the interdependencies between natural systems (e.g. climate systems) and community health and well-being [4,5] and is likely to be significantly influenced by climate change. There is an intrinsic relationship between the health of ecological systems, health of communities and the health of people. As climate change continues, for example: the frequency, intensity and duration of heatwaves will increase; the distribution of mosquitoes and other insects will shift; sea level and ocean acidity will continue to rise; and food production and water resources will be affected. All these factors will have direct or indirect impacts on population health [3,6]. The 2003 European heatwave, hurricane Katrina in the United States in 2005, and the Melbourne bushfires in 2009 all caused increased mortality/morbidity and severe damage to communities [7-9] which may be regarded as direct impacts from our changing climate.

Other climate change-related impacts on eco-environmental health however, are more indirect and subtle. For example, climate change can negatively impact on food production, water quality and quantity, air quality, ecosystem functions [10-12]. These temporal and geographical variations in weather and climate are harmful to eco-environmental health causing an indirect impact on human health and well-being [13-15]. Furthermore, climate change will lengthen the transmission season for vector-borne diseases in some areas, posing a significant threat to human health. For example, an increase in mean temperatures has been observed to increase the geographic distribution of mosquitoes and to shorten the incubation period of the pathogen within the vector [16], which is likely to increase the risk of vector-borne diseases such as malaria, Dengue fever, Lyme disease, and Ross River Virus [17-20].

In the work of estimating the impacts on eco-environmental health from climate change, the vulnerability of communities and individuals is a key issue that should be examined further [21]. A typical definition of vulnerability is *susceptibility to be harmed *[22]. The IPCC defines vulnerability as the degree to which a system is susceptible to and is incapable of coping with the adverse effects of climate change [3]. Human health vulnerability to climate change is also defined as a function of a) sensitivity; b) the exposure to the climate-related hazard; and c) the adaptation measures in place to reduce the adverse health outcomes [23]. Factors such as age, location, social connectedness, culture and social and economic resources are also important in the vulnerability assessment of climate change [24-26].

It still remains unclear however, as to how the vulnerability of eco-environmental health to climate change can be best assessed and which framework should be adopted for this purpose. Thus, in order to devise and implement adaptation strategies to climate change, it is important to develop a consistent set of definitions and frameworks for assessing eco-environmental health vulnerability. Such a framework can help ensure that the process of examining, interpreting and representing vulnerability is done systematically, thus reducing the likelihood of analytical inconsistencies. It may improve the communication of methods and results of vulnerability assessments; encourage systematic assessments; and facilitate the translation of research into policy and practice [27].

Governments and international agencies are increasingly concerned with the potential eco-environmental consequences of emerging issues such as climate change. Given its possible serious current and future impacts, assessments of eco-environmental health vulnerability to climate change are urgently needed to assist governments in the development of adaptation strategies, policies, and measures to lessen the projected adverse impacts on eco-environmental health. Even though a few frameworks for vulnerability assessments have been developed and implemented [23,28,29], none of them has attempted to quantify the level of interdependencies between ecological systems and population health and to directly link the research findings with social and public health policies. Our aim was therefore to examine perceptions and views from government stakeholders and other relevant specialists about the threat of climate change, the capacity to deal with it, and how to develop and implement a framework for assessing vulnerability of eco-environmental health to climate change.

## Methods

### Design and Setting

This study is the qualitative component of a three-year project aiming to develop a framework for assessing vulnerability of eco-environmental health to climate change. The qualitative component was based on a focus group methodology in order to investigate attributes relevant to vulnerability of eco-environmental health of climate change. The use of focus groups in qualitative research is useful when seeking in-depth knowledge of this nature [30]. Two focus group discussions with 7-8 people per group, which is the desirable size for groups consisting of knowledgeable participants and dealing with complex issues [31], were conducted. The focus groups consisted of representatives from relevant government agencies, industry, and non-governmental organisations to facilitate an interdisciplinary team providing breadth and depth to the discussions. The focus groups took place at the Queensland Government's Department of Environment and Resource Management office complex in Brisbane, Australia. The first focus group was conducted on 10 December 2008 and the second on 3 June 2009. All of the participants were fully informed about the purpose and process of this study and gave written consent to participate prior to the focus group discussions.

### Sampling

The approach used for recruiting participants in this study was to invite, through phone calls and emails, groups of professionals working within environmental protection, climate change and public health. In order to gain sufficient insight into the knowledge base and interdisciplinary expertise available in Brisbane, we sought to have enough participants to conduct two focus groups. The participants were primarily from relevant government agencies (n = 11) including Queensland Department of Community Safety, Department of Health, Department of Main Roads, Department of Environment and Resource Management, Ambulance Services, Office of Climate Change; and non-governmental organisations (n = 2) including the Clean Air Society of Australia and New Zealand (Table 1). The participants were purposely selected from these agencies because they are involved in the areas of climate change, natural disasters and public health and are responsible for climate change and public health policy and planning. To add additional breadth and depth to the discussions we included participants from the industry sector (n = 2) such as Queensland Mining Industries.

**Table 1:** Characteristics of the participants

Specialists	Female	Male	Total
Department of Environment and Resource Management	0	3	3
University Scientists	0	2	2
Queensland Health	0	2	2
Industry Specialists	1	1	2
Clean Air Society of Australia and New Zealand	0	2	2
Department of Infrastructure and Planning	0	1	1
Department of Community Safety	0	1	1
Office of Climate Change	1	1	2

**Total**	**2**	**13**	**15**

### Data Collection

A protocol was developed before the focus groups were conducted. Open questions were used to facilitate in-depth discussion. Several key issues relevant to vulnerability and adaptive capacity were discussed, including the major factors perceived to influence the vulnerability of eco-environmental health to climate change and how to minimise the impact of climate change on eco-environmental health. The key questions asked include:

• Should people worry about climate change? If so, why?

• Do you think climate change will affect eco-environmental health?

• Is it important to assess the vulnerability of eco-environmental health to climate change? If so, why?

• What concerns do you have when assessing the vulnerability of eco-environmental health to climate change?

• In your view, how can the vulnerability of eco-environmental health to climate change be assessed?

• In the absence of assessment, how can the impacts of climate change on eco-environmental health be minimised?

• To what extent can adaptation to climate change reduce the vulnerability?

• How can we identify a framework for assessing the vulnerability of eco-environmental health to climate change?

• What are the key aspects of climate change to consider?

• What strategies would you propose to implement the framework at different levels of government?

### Data Analysis

Data from the focus group discussions were transcribed and independently checked. Contemporaneous notes were also taken during the meetings to assist with the transcription. The analyst familiarised herself with the data by reading and re-reading the transcripts and written notes. In order to derive themes and key points from the data, a framework approach was used [32]. The final stage of this approach - mapping and interpretation - was conducted according to the recommended eight criteria in qualitative research: *words, context*, *internal consistency, frequency, intensity of comments, specificity of responses, extensiveness and big picture *[32].

## Results

Six main themes stood out from the transcripts and notes. These themes were: the threat of climate change, perceptions about implications, identification and assessment of vulnerability, factors that influence vulnerability, minimising the impact, and development and implementation of a framework (Table 2).

**Table 2:** Major findings from the focus group discussions

Main themes	Major findings
The threat of climate change	The majority thought that our climate is truly changing but some were sceptic
	More extreme weather events
	Important to act before it is too late

Perceptions about implications	Direct and indirect impacts are already happening
	Drought, bushfires, and vector-borne diseases were brought up as examples
	Not only was the temperature increase but also climate variability perceived as concerning

Identification and assessment of vulnerability	Important to know what you are measuring
	Hazard characterization by dividing it into acute and chronic hazards
	Communities can decrease their vulnerability through adaptation

Factors that influence vulnerability	Population growth
	Ageing population
	Coastal development
	Global economic crisis

Minimising the impact of climate change on eco-environmental health	Mitigation was perceived as important
	Adaptation measures (e.g., insulation of houses)
	Public education
	Preparedness in case of extreme weather events

Development and implementation of a framework	Review of government documents
	Examination of existing frameworks
	Assessment on how Queensland Government has responded to previous extreme weather events

### The Threat of Climate Change

There was some disagreement among the participants in the focus groups about whether climate change is really happening or not. One participant stated that he was 'sceptical' about climate change.

'...in my lifetime lots of things have happened and I find that the thing that is new is that they are being attributed to climate change'

Another participant remarked that there used to be more cyclones and extreme weather in the early 70 s. The fact that Queensland always has had extreme events like floods and bushfires was also emphasised with another participant expressing concerns regarding the fact that extreme events, now attributed to climate change, have always been occurred. This view was challenged however, with other participants pointing out recent extreme events -such as the 2004 record heatwave in Brisbane and the ongoing drought throughout much of southern Queensland that according to the participant is the worst drought in Brisbane since early 1900.

The majority of the participants agreed on the fact that our climate is changing. A general concern was that increasing temperatures and greater variability pose significant threats to eco-environmental health. The participants all agreed on the reality that our cities are unsustainable and that this leads to degradation of our environment. One participant noted:

'We are pushing the limits of sustainability'

Lastly, precautionary principles were mentioned and the importance of acting before it is too late was emphasised:

'...the evidence the next ten years will show us whether we can relax or if we should do even more but I don't think that we can pause the decisions until we get more evidence....'

### Perceptions about Implications

During both focus groups, a rich discussion was focused on direct implications such as more frequent and intense extreme weather events and their effects on eco-environmental health. Heatwaves were brought up as an example:

'We do have about 10,000 deaths each year in Europe as a result of heatwaves'

Some participants expressed that they did not think that climate change would necessarily affect human health directly, but that it would be a subsequent threat to human health from damages made to the environment. Impacts from increased temperatures on the ocean temperature and its subsequent effect on the coral reefs were mentioned as an example to support this statement. Furthermore, the impact of drought on food and water supplies was emphasised. There was also general concern about mosquito breeding and the impact of climate change on vector-borne diseases. Some participants were concerned that millions die from Malaria every year and as the temperature increases, the disease may spread to new areas that were previously free of this disease. One participant described this particular issue as follows:

'Climate change affects the natural environment in providing extra breeding grounds for mosquitoes for example. The temperatures and water reservoirs for mosquitoes breeding, which can then lead to increase in the mosquito numbers which can then lead to people getting exposed which can then lead to mosquito-borne diseases'

Several participants expressed climate *variability *as a more important concern in regard to eco-environmental health. The variability may exhibit more harm to eco-environmental health than the actual average temperature.

### Identification and Assessment of Vulnerability

Assessment of vulnerability was perceived to be of key importance as it will help prepare for the future implications of climate change. One participant highlighted the importance of knowing what you are measuring and another emphasised the importance of looking at which parameters are used for the purpose of assessment. For example, when life-expectancy is used as a measure of health status in a country, it may misleadingly be interpreted as a sign of resilience because of an apparent reduced *sensitivity *in the population. However, the population could still be very vulnerable to climate change due to other factors such as high exposure and a lack of adaptation measures being in place.

Characterising climate change in terms of natural hazards was one suggested method. One participant divided the threat from climate change into two categories, classified as acute and chronic climate hazards. Chronic natural hazards, such as drought and soil salinisation, would be slower and more difficult to identify than acute natural hazards. Variability and extreme weather events such as floods and cyclones were considered acute natural hazards. The need to assess which of the anticipated natural hazards are chronic hazards and which are acute were emphasised as important in order to respond correctly to them.

'there is a bit of breaking down of that problem to get an understanding of which are acute events and which are the chronic ones that need to be focused on because the response strategies will be quite different'

One participant however, pointed out the complexity of assessment of vulnerability when multiple hazards occur simultaneously:

*'I suppose an example could be something like that we had a tropical cyclone passing the coast where we had high winds, localised fires due to rain depressions, we had storm surges coming through*. *To be able to identify vulnerability to those as individual hazards is relatively easy, but then adding all of those three together adds a little complexity to the assessment'*

Some participants believed that it is not vulnerability to the hazard that changes, but rather the frequency and severity of the hazard. An individual's vulnerability was perceived as constant as long as that person lived in the same house, earned the same money and was surrounded by the same networks throughout his/her life. Communities, on the other hand, were perceived as capable of decreasing their vulnerability if taking the right measures. Potential community adaptation strategies include developing emergency management response plans, having adequate emergency shelters in place, community awareness, business continuity planning, risk management, early warning systems, well-known evacuation routes and assembly points, emergency stores, and partnerships between local government and small businesses.

### Factors that Influence Vulnerability

A range of social, physical and economic factors were identified by participants as important considerations when assessing vulnerability. Population growth was identified as a key factor which may lead to higher demand on valuable resources, such as water supplies, thereby increasing the vulnerability of the population. Some participants were concerned about the ageing of the population which was perceived as having a significant impact on the vulnerability of eco-environmental health to climate change. The increasing proportion of elderly people and the associated health problems among the elderly were understood as factors decreasing the adaptive capacity in a community.

Also, physical changes to the landscape such as increasing and expanding coastal development in vulnerable areas are adding to the vulnerability of the Queensland population. One participant stated:

'If you look at the age profile for Queensland and for South-East Queensland in particular, you might argue that even if we had the current level of hazard, and the current frequency and intensity of climate events, the extra coastal development and the changes in our population structure, you might say, are enough for concern independent of climate change'

Another participant was concerned about the canal-style development that is a feature of coastal housing developments especially along the South-East Queensland coastline. Due to the properties' proximity to the water, this development makes the households particularly vulnerable to flooding.

Finally, discussions were centred on the current global financial crisis with people losing their jobs and their homes, reducing their ability to deal with disasters and increasing their vulnerability to climate change.

### Minimising the Impact of Climate Change on Eco-environmental Health

Mitigation, meaning measures to reduce emissions to slow down or reverse climate change, was briefly discussed in both focus groups. One participant noted that people should be encouraged to minimise their carbon emissions (e.g. to use public transport instead of cars) and exploration of new sources of renewable energy was emphasised. In order to achieve these goals however, collaboration between governmental agencies and the importance of sharing information between sectors about the benefits of for example, building sustainable housing and infrastructure, were emphasised. Such inter-sectoral collaboration was perceived as crucial to reduce greenhouse gas emissions.

In terms of adaptation measures, there was broad agreement about the fact that Queensland is "well off" because of its highly developed society and advanced economy increasing its adaptive capacity. In terms of adaptation strategies, some participants mentioned that it is necessary to assess emergency responses and to increase preparedness.

'...understanding things like where our emergency shelters are now, if we did get that cyclone, do all the people in South-East Queensland know where they are? Have they got evacuation plans? That would be a good place to start' 

The participants also highlighted the importance of making sure that people are aware of the problem and are provided with sufficient education on for example, how to minimise their emissions and what to do in case of natural disasters. Also, precautionary measures such as food and water safety and supplies were perceived as important in terms of preparing for potential future extreme weather events. There is much people can do to prepare themselves for extreme weather events due to climate change. Social networks were mentioned as an important component within this context. Elderly people living alone in particular, need friendly neighbours who care and check up on them to make sure that they are safe during extreme weather events such as heatwaves.

Some participants highlighted that policy issues in regard to the built environment needed addressing. For example, while it is recognised that air-conditioning reduces mortality, it also increases our energy consumption and therefore greenhouse gas emissions. Building codes should include insulation of new houses which helps keep the temperature more constant through more environmentally friendly means.

### Development and Implementation of a Framework

Participants suggested that a comprehensive review of government documents needs to be undertaken which focuses on frameworks for vulnerability assessment. Governmental departments involved with the assessment of vulnerability should also be engaged in developing a suitable framework. One participant also suggested that priority should be given to defining the institutional understanding of this issue.

One participant argued that there is no real difference between vulnerability and risk. However, most participants agreed on the fact that risk is the frequency and severity of the hazard and that vulnerability is the sensitivity of different population groups to these risks. Others thought that vulnerability involves at least two separate components - risk and adaptation. One participant explained that:

'...some people are confused about the difference between the two terms. Risk and vulnerability. But in my view I think there is a distinction between risk and vulnerability. Vulnerability should take both risk and adaptation into account. So vulnerability is not equal to risk'

Some participants thought that already existing frameworks should be examined and used as a basis when developing our own. Different hazards require different tools. There was agreement within one focus group that in a framework for assessing vulnerability of eco-environmental health to climate change, an important aspect would be to look at the impact of storms, bushfires and heatwaves that have previously occurred in Queensland. In regard to implementation of such a framework, all participants agreed that it should be implemented at the three levels of government - commonwealth, state and local.

## Discussion

Through two focus groups, we examined perceptions from government stakeholders and other relevant specialists about the threat of climate change, perception about implications, identification and assessment of vulnerability, factors that influence vulnerability, minimising the impact, and development and implementation of a framework for assessing vulnerability of eco-environmental health to climate change. The data retrieved from the transcripts of the focus group discussions showed that despite a few sceptics, the participants were well aware of the threat climate change poses to eco-environmental health. They acknowledged the importance of vulnerability assessment and identified a range of factors influencing eco-environmental health vulnerability. They also had a rich discussion on both mitigation and adaptation measures and seemed to have a certain amount of knowledge about how to minimise the impacts of climate change on eco-environmental health. Findings from the study however, do suggest that the participants were unable to contribute substantially to the development of such a framework. Thus, work needs to be done to promote further understanding of many factors that need to be considered when developing a framework assessing the vulnerability of eco-environmental health to climate change.

The results of this study are consistent with two previous studies undertaken on public health officers and health department directors in the United States [33-35]. These studies found that a majority of the surveyed health department directors and public health officers perceived climate change to be a relevant threat in their respective areas. The majority of the participants in the current study believe that our climate is changing and that something needs to be done to protect eco-environmental health. As confirmed by previous quantitative studies [36-38], the participants used the adverse effects on eco-environmental health from heatwaves as an example and referred to the recent heatwave event experienced in Queensland. The participants perceived an assessment of vulnerability as important and mentioned factors such as chronic and acute hazards, the complexity of multi-hazard assessment and the capacity of communities to protect themselves from the impact of climate change as key considerations within vulnerability assessment. It may prove to be problematic however to divide 'hazards' into chronic and acute hazards; as the hazards defined as chronic (e.g., drought) not only have chronic effects, but also may lead to acute incidents such as suicide committed by distressed farmers [39]. Nevertheless, professionals within public health can play a important role in reducing eco-environmental health vulnerability to climate change through promotion of "healthy people, healthy homes and healthy communities" [40].

Factors influencing vulnerability were discussed and population growth and coastal and canal development were identified as causes for concern among the stakeholders. Low-lying land (less than 10 meters above sea level) covers 2 per cent of the world's land area and is home to 10 per cent of the world's population and 13 per cent of the world's urban population [41]. Given the estimated sea-level rise at different scenarios of between 0.18 and 0.59 meters by 2100 [3], this becomes an important population group when assessing vulnerability of eco-environmental health to climate change. The vulnerability of eco-environmental health was also perceived to increase with an increasing elderly population, exemplified by the death rate observed among the elderly population following hurricane Katrina. The death rate for this group in New Orleans was more than 15 times that of the non-elderly population [7].

The participants discussed mitigation measures such as decreasing emissions, renewable energy and emphasized the responsibility of government sectors other than health and the importance of inter-sectoral collaboration. They also acknowledged that Australia has the economy and the resources to develop and implement adaptation measures. However, a perceived lack of awareness among the general population of the implications of climate change for public health was also suggested by the participants. If this is the case, it may have implications for policy making and implementation. It is clear that this issue needs to be prioritised when devising new policy because of problems that might arise from lack of public support during the stage of policy and program implementation.

In terms of developing a framework to assess vulnerability of eco-environmental health to climate change, there has to date been little qualitative research on the perceptions of public health specialists and climate change specialists. The participants in our study suggested that an important aspect that needs to be considered when developing a framework is examination of the previous impacts of for example, storms and bushfires. This might be more relevant however, to an actual vulnerability assessment than it is to developing an assessment framework. These findings suggest that we need to work more closely with government decision makers in the development of an eco-environmental health framework to ensure that their needs are well met and their concerns are properly addressed. In addition, this study demonstrates that some people who are positioned to contribute to the development of implementation of strategies aimed at protecting eco-environmental health from threats posed by climate change do not believe in climate change. This suggests that there is a clear need to improve the communication between scientists and decision makers to ensure that they understand fully the need to consider potential threats from climate change, regardless of their personal beliefs.

Although this study is local and focuses on the climate change implications for South-East Queensland and the Brisbane area, the research findings may have some implications for other industrialised countries. Even though most developed countries may have the necessary resources to meet the present and future challenges arising from the changing climate, there may still be some population groups at risk such as people living in coastal zones and the elderly who are especially vulnerable to the impacts of climate change. This conclusion is supported by findings from a study undertaken in Norway where vulnerability was found to emerge within some regions, localities, and social groups [42]. In order to deal with the challenges of climate change and climate variability, it will therefore be necessary to recognise vulnerability to climate change at different levels (local and regional), and to address them accordingly.

A number of limitations associated with this study warrant attention. As the sample was small and discussion time was limited, it is unlikely to cover the perceptions of public health and climate change specialists on this topic in a comprehensive way. While the semi-structured interview format of the focus groups may have enhanced the quality of data obtained through participant interaction, it may also have reduced responses by participants who were less confident about sharing their perspectives in a group setting compared to that provided by a one-on-one interview approach, thereby discouraging those with different perspectives from speaking up [43]. The fact that we undertook two focus groups on the subject did however ensure that conclusions drawn from this study were based on a range of information gathered from a range of specialists across two groups, thus increasing the chances of different perspectives being represented, heard and documented. Also, the fact that only two participants were from public health authorities may have limited this study's ability to reflect the overall knowledge about the impacts of climate change on eco-environmental health. Lastly, researchers were unable to explore some issues in depth (for example, how to develop a framework) due to time constraints of the focus group sessions.

## Conclusions

Despite the limitations outlined above, this study has addressed some important questions in regard to public health and climate change specialists' views on the threat of climate change and its implications. The government stakeholders and specialists in the present study appeared to have considerable knowledge on the impacts from climate change on eco-environmental health, and they pointed out important factors influencing vulnerability and identified several vulnerable population groups. These qualitative findings may have implications in climate change and public health decision-making.

## Competing interests

The authors declare that they have no competing interests.

## Authors' contributions

LBS took notes during the focus group discussions, transcribed the audio tapes, carried out the data analysis, and produced the final manuscript. ST was responsible for the design and coordination of the study, acted as the facilitator at the focus groups and helped prepare the manuscript. RA helped with the qualitative part of the manuscript and revised it. DM read and revised the manuscript. All authors read and approved the final manuscript.

## Pre-publication history

The pre-publication history for this paper can be accessed here:

http://www.biomedcentral.com/1471-2458/10/441/prepub
